# Pattern of Drug Resistance and Risk Factors Associated with Development of Drug Resistant *Mycobacterium tuberculosis* in Pakistan

**DOI:** 10.1371/journal.pone.0147529

**Published:** 2016-01-25

**Authors:** Irfan Ullah, Arshad Javaid, Zarfishan Tahir, Obaid Ullah, Aamer Ali Shah, Fariha Hasan, Najma Ayub

**Affiliations:** 1 Department of Microbiology, Faculty of Biological Sciences, Quaid-i-Azam University, Islamabad, Pakistan; 2 Programmatic Management of Drug Resistant TB Pulmonology, Lady Reading Hospital Peshawar, Pakistan; 3 Department of Bacteriology, Institute of Public Health, Jail Road Lahore, Pakistan; Indian Institute of Science, INDIA

## Abstract

**Background:**

Drug resistant tuberculosis (DR-TB) is a major public health problem in developing countries such as Pakistan.

**Objective:**

The current study was conducted to assess the frequency of drug resistant tuberculosis including multi drug resistance (MDR- TB) as well as risk factors for development of DR-TB, in Punjab, Pakistan.

**Methodology:**

Drug susceptibility testing (DST) was performed, using proportion method, for 2367 culture positive *Mycobacterium tuberculosis* (MTB) cases that were enrolled from January 2012 to December 2013 in the province of Punjab, Pakistan, against first-line anti-tuberculosis drugs. The data was analyzed using statistical software; SPSS version 18.

**Results:**

Out of 2367 isolates, 273 (11.5%) were resistant to at least one anti-TB drug, while 221 (9.3%) showed MDR- TB. Risk factors for development of MDR-TB were early age (ranges between 10–25 years) and previously treated TB patients.

**Conclusion:**

DR-TB is a considerable problem in Pakistan. Major risk factors are previous history of TB treatment and younger age group. It emphasizes the need for effective TB control Program in the country.

## Introduction

Tuberculosis is a common infectious disease of human beings, mainly caused by *Mycobacterium tuberculosis* MTB [1]. Multi-drug resistant tuberculosis (MDR-TB) is a serious problem where the bacterium is resistant to at least two of the most powerful first-line anti-TB drugs such as Rifampicin (RIF) and Isoniazid (INH) with or without any other drug. Globally, 3.7% of the newly diagnosed and 20% of the previously treated cases for TB were estimated to have MDR-TB in 2012 [2]. The highest level of MDR-TB are found in Central Asia and Eastern Europe where in certain countries more than 50% of previously treated and more than 20% of new TB cases have MDR-TB. Almost 60% of MDR-TB in newly diagnosed cases have been reported from China, Russian Federation and India [2]. Extensively drug resistant (XDR) TB {isolates as MDR with additional resistance to fluoroquinolone and any second line injectable drugs (amikacin, capreomycin and/ or kanamycin),} has now been reported in 92 countries, and an estimated 9.6% of the MDR-TB cases have developed XDR-TB [2].

Pakistan has been ranked 5^th^ in 22 high TB burden countries and 4^th^ among countries where MDR-TB has become a serious challenge for clinicians [3]. The overall prevalence of TB cases in Pakistan has been estimated as 342 per 100,000 population [4]. WHO estimated MDR-TB in new cases is 3.5% and 21% in the previously treated TB cases [4]. Lack of effective TB control programme in the country in the past, illiteracy and poverty are among the main reasons of high prevalence of drug resistant tuberculosis in populations. Objective of the present study was to determine the frequency of drug resistance (DR) to first-line anti-TB drugs and risk factors for development of drug resistance in the Punjab, largest province of Pakistan with population of 101 million and area of 205, 344 km^2^.

## Materials and Methods

This study was approved by research and ethics committee of Quaid-i-Azam University, Islamabad, Pakistan. Written consents were obtained from all patients or from next of their kin, caretakers, or guardians.

Six thousand and six specimens collected from suspected TB patients at department of bacteriology, institute of public health, Lahore, Punjab province, Pakistan from January 2012 to December 2013 were included in this study. The referrals of these specimens were according to the guidelines of National TB Control Programme (NTP) for referral of specimens for culture.

This was a cross sectional descriptive study. Patients with a history of cough for more than two weeks and/or pulmonary X-Ray abnormalities of all age groups, genders, newly suspected or previously treated (i.e. relapse, failure, default) and patients taking anti-TB treatment at the time of study, were recruited for the study. A TB suspect was interviewed for medical and treatment history that included physical examination, personal information, current illness, TB related complaints, previous personal and family medical history. The treatment history included present and previous TB treatment if any.

All specimens were decontaminated and processed for culture as per standard protocol [5]. All the confirmed MTB isolates from positive cultures were subjected to drug sensitivity test (DST). All MTB isolates on Lowenstein-Jensen (LJ) medium were identified by using *p*-Nitro benzoic acid (PNB) method [6,7]. *Mycobacteria* other than tuberculosis (MOTT) were excluded from the final study. Drug susceptibility testing was performed for rifampicin (40 μg/ml), isoniazid (0.2 μg/ml), streptomycin (4 μg/ml), ethambutol (2 μg/ml) and pyrazinamide (100 μg/ml) using 1% proportion method as per the standard guidelines [8] and as described by Canetti *et al*. [9]. *Mycobacterium tuberculosis* H37RV isolate, which is characteristically susceptible to all anti-TB drugs, was used as a reference control strain.

### Statistical analysis

Association between categorical variables was observed by using Chi square test (and Fisher exact wherever necessary). The criterion for significance was set at P<0.05 based on a two-sided test. When significant association was observed upon Chi square test, Phi and Cramer values were calculated to observe the direction and strength of association. Phi, Cramer value of <0.1 was considered as negligible association, whereas Phi and Cramer value of 0.1–0.2, 0.201–0.4 and >0.4 was considered as weak, moderate and strong association, respectively. The data was analyzed by using Statistical Package for Social Sciences (SPSS 18).

## Results

**Fig 1:**
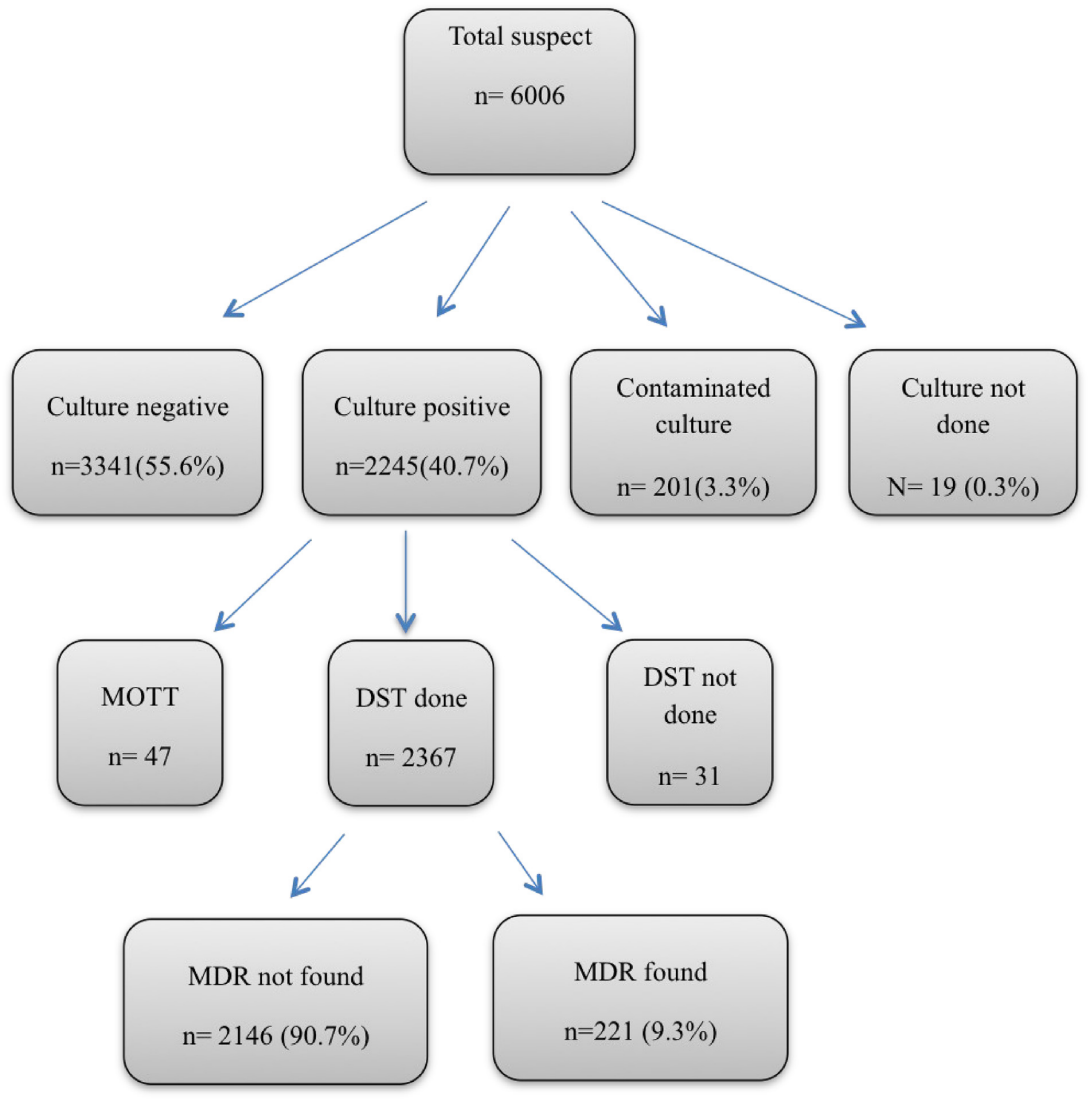
Flow chart of specimens’ over all processing for investigating MDR-TB isolates.

Out of 6006 suspected specimens cultured, 2445 (40.7%) were positive, 3341 (55.6%) were negative, 201 (3.3%) were found contaminated and culture could not be performed on 19 (0.3%) cases due to poor quality of specimens. Among 2445 positive culture specimens, 47 were found to be MOTT and DST could not be applied on 31 cases. Culture negative specimens, contaminated, MOTT and those on which DST could not be performed were excluded from the study. Thus 2367 patients were included in the final analysis (Fig 1). The mean ages of patients were 39.7+ 18.5 years, 1238 (52.3%) of them were female and 1129 (47.7%) were male, 1566 (66.2%) were new and 801 (33.8%) were previously treated patients (Table 1).

**Table 1:** Association between patients’ demographics and drug susceptibility testing results.

Variables	Drug resistance pattern					MDR
	Rifampicin (%)	Isoniazid (%)	Streptomycin (%)	Ethambutol (%)	Pyrazinamide (%)	
**Gender**						
Female	127 (10.3)	123 (9.9)	35 (2.8)	27 (2.2)	19 (1.5)	117 (9.5)
Male	114 (10.1)	110 (9.7)	35 (3.1)	38 (3.4)	20 (1.8)	104 (9.2)
**P-value**	0.47	0.57	0.71	0.04	0.52	0.84
**Phi value**	-	-	-	0.05	-	-
**Age (years)**						
10–25	110 (11.2)	114 (11.6)	32 (3.2)	19 (1.9)	13 (1.3)	108 (11.0)
26–45	84 (10.6)	76 (9.7)	20 (2.5)	28 (3.5)	16 (2)	74 (9.4)
46–65	39 (8.7)	37 (8.3)	13 (2.9)	13 (2.9)	9 (2)	35 (7.8)
>65	8 (5.5)	6 (4.1)	5 (3.4)	5 (3.4)	1 (0.7)	4 (2.8)
**p-value**	0.12	0.06	0.83	0.37	0.68	0.008
**Phi value**	-	-	-	-	-	0.07
**TB treatment History**						
New patients	74 (4.7)	68 (4.3)	30 (1.9)	13 (0.8)	15 (1)	63 (4.0)
Previously treated patients	167 (20.8)	165 (20.6)	40 (5)	52 (6.5)	24 (3)	158 (19.7)
**p-value**	<0.001	<0.001	<0.001	<0.001	<0.001	<0.001
**Phi value**	0.25	0.26	0.08	0.16	0.07	0.255

Among 2367 cases, rifampicin resistance was present in 241 (10.2%) cases, isoniazid resistance in 233 (9.9%), streptomycin resistance in 70 (2.9%), ethambutol resistance in 65 (2.7%), pyrazinamide resistance in 39 (1.6%) and 221 (9.3%) were found to have MDR-TB. Upon Chi square analysis no statistical significant association was observed between patients’ gender and MDR-TB. Gender wise drug resistance pattern given in Table 1.

Among MDR- TB cases, comparison has been done between age groups 10–25, 26–45, 46–65 and above 65 years. Age group 10–25 years was the most affected group with MDR-TB as compared to others age groups. Upon Chi square analysis statistically significant negligible positive association was observed between patients aged 10–25 years and DR-TB (P<0.008, φ = 0.07). The drug resistance pattern of age group given in Table 1.

Among the new cases, drug resistance was found in 200 (12.8%) while MDR-TB in 63 (4%) patients. Out of previously treated patients drug resistance was observed in 448 (56%) and MDR-TB in 158 (19.7%). When we cross tabulated TB treatment history with MDR-TB prevalence, statistically significant moderate positive association was observed between previous TB treatment and MDR-TB (P<0.001, φ = 0.25). MDR-TB cases were 5 times more prevalent among previously TB treated than in newly diagnosed cases (OR 9.582, 7.12–12.66, P<0.001, φ = 0.255) (Table 1).

The univariate analysis revealed a higher proportion of female gender (cOR 1.026; 95% CI 0.798–1.319), individuals in age group 10–25 years (OR: 1.560; 95% CI 1.118–2.177) and previously treated patients (OR: 5.862; 95% CI (4.316–7.963) was found amongst MDR-TB patients. The multivariable logistic regression model identified female gender (aOR: 1.004; 95% CI: 0.753–1.338), of age group 10–25 years (aOR: 0.607; 95% CI: 0.420–0.878) and previously treated patients (aOR: 5.879; 95% CI: 4.326–7.990) to be associated with MDR- TB (Table 2).

**Table 2:** Factor associated with multi drug resistant TB.

	Non-MDR, 2146 n (%)	MDR-TB, 221 n (%)	Univariate			Multivariate		
			cOR	95%CI	p- Value	aOR	95%CI	p- Value
Female gender	1121 (52.2)	117 (9.5)	1.026	0.798–1.319	0.842	1.004	0.753–1.338	0.981
10–25 years	1592 (74.1)	182 (82.2)	1.560	1.118–2.177	0.008	0.607	0.420–0.878	0.008
Previous TB treatment	643 (30)	158 (19.7)	5.862	4.316–7.963	<0.001	5.879	4.326–7.990	<0.001

cOR, crude ratio; aOR, adjusted odds ratio, adjusted for gender, age and treatment history.

## Discussion

Here we presented the results of a large number of TB sample collected from different arrears of the Punjab Province, Pakistan. In TB endemic country like Pakistan, 9.3% of MDR-TB is in the line with other reported studies from within the country [10]. Previous TB treatment was found to have statistically significant association with culture positive results. In our study, although more new patients were suspected for TB than the previously TB treated patients; however, in past, TB was more common in previously treated patients [11]. This study found that the frequency of drug resistance in previously treated TB is 5 times higher than those of newly diagnosed patients for a single drug as well as for all first line anti-TB drugs, as stated earlier in other studies [12,13]. Increase in resistance to anti-TB drugs against MTB causes significant threat to TB control [14, 15].

Among the resistant cases in this study, the prevalence of MDR-TB in newly diagnosed and previously treated patients was 4% and 19.7%, respectively, which is similar to the previously reported studies [4,16–21]. However, MDR-TB has also been reported to be different in different geographical areas. The prevalence of MDR-TB in new and previously treated cases in Japan has been reported as 0.7% and 9.8% respectively [22], from China 2.8 and 14.7% and in another study 9.7 and 34.3% in new and previously treated cases, respectively [23, 24] and from Brazil Micheletti et al. reported the overall MDR-TB as 4.7%, in newly diagnosed was 2.2% and in previously diagnosed patients was 12% [25]. These differences may be due to different levels of health care delivery system in various countries, TB Control Programme, living standards, and socio- economic factors. The resistance in previously treated cases is indicator of poor compliance, lack of treatment supervision and ineffective TB Control Programme whereas in new cases is due to the transmission of disease with resistant bacilli [26].

Upon Chi square analysis no statistical significant association was observed between patients’ gender and MDR-TB in the current study. However, there were conflicting results in the literature about vulnerability of female gender to MDR-TB [19, 27]. Mor et al. stated that males are more at risk for MDR-TB [28].

Age was also found as an important factor in the drug resistance development. In this study more patients in the age group 10–25 years developed MDR-TB. Similar findings were observed while reviewing the previously published reports [18,29,30]. In the literature there is no well-established association between age and the risk of MDR-TB because different studies used different age group cut-off points. The age related difference in compliance may be a possible reason, as patients in the age group 10–25 years are often busy in activities like education, work or other activities on a daily basis, in comparison to more inactive lifestyle of older age patients [30].

## Conclusion

Drug resistant TB was found to be very high in the province of Punjab, Pakistan. The major risk factor for development of drug resistant TB was history of previous TB treatment. This fact emphasizes the need for properly functioning TB Control Programme with strict supervision of patients ensuring compliance and completion of treatment. MDR-TB was present five times higher in previously TB treated as compared to newly diagnosed patients. Prevalence of rifampicin and isoniazid drug resistance in the studied patients was higher as compared to other first line drugs. Moreover, early age group 10–25 years was considered to be mostly affected both in males and females with MDR-TB.
